# Abordaje del uso y las complicaciones de las siliconas líquidas inyectables en población travesti y trans: experiencias de profesionales de la salud de Argentina

**DOI:** 10.18294/sc.2025.5202

**Published:** 2025-02-19

**Authors:** Constanza Lupi, Daniela Paola Bruno, Diego Daniel Salusso, Estefania Panizoni, Lara Weitz, Cecilia Valeriano, Inés Aristegui

**Affiliations:** 1 Especialista en Planificación y Gestión de las Políticas Sociales. Coordinadora, Dirección de Programas, Fundación Huésped, Ciudad Autónoma de Buenos Aires, Argentina. constanza.lupi@huesped.org.ar Fundación Huésped Fundación Huésped Ciudad Autónoma de Buenos Aires Argentina constanza.lupi@huesped.org.ar; 2 Doctora en Ciencias Sociales. Investigadora, Instituto de Investigaciones Gino Germani, Facultad de Ciencias Sociales, Universidad de Buenos Aires, Ciudad Autónoma de Buenos Aires, Argentina. danielapaolabruno@gmail.com Universidad de Buenos Aires Instituto de Investigaciones Gino Germani Facultad de Ciencias Sociales Universidad de Buenos Aires Ciudad Autónoma de Buenos Aires Argentina danielapaolabruno@gmail.com; 3 Médico Especialista en Enfermedades Infecciosas. Investigador Clínico, Fundación Huésped. Docente adscripto, Facultad de Medicina, Universidad de Buenos Aires. Ciudad Autónoma de Buenos Aires, Argentina. diego.salusso@huesped.org.ar Universidad de Buenos Aires Facultad de Medicina Universidad de Buenos Aires Ciudad Autónoma de Buenos Aires Argentina diego.salusso@huesped.org.ar; Fundación Huésped; 4 Médica especialista en Clínica Médica. Investigadora Social, División de Investigación en Implementación, Fundación Huésped, Ciudad Autónoma de Buenos Aires, Argentina. estefania.panizoni@huesped.org.ar Fundación Huésped División de Investigación en Implementación Fundación Huésped Ciudad Autónoma de Buenos Aires Argentina estefania.panizoni@huesped.org.ar; 5 Diplomada en Políticas de Salud en el Territorio. Dirección de Programas, Fundación Huésped, Ciudad Autónoma de Buenos Aires, Argentina. lara.weitz@huesped.org.ar Fundación Huésped Fundación Huésped Ciudad Autónoma de Buenos Aires Argentina lara.weitz@huesped.org.ar; 6 Diplomada en Tratamiento Comunitario. Directora de Programas, Fundación Huésped, Ciudad Autónoma de Buenos Aires, Argentina. cecilia.valeriano@huesped.org.ar Fundación Huésped Fundación Huésped Ciudad Autónoma de Buenos Aires Argentina cecilia.valeriano@huesped.org.ar; 7 Doctora en Psicología. Directora, División de Investigación en Implementación, Fundación Huésped, Ciudad Autónoma de Buenos Aires, Argentina. ines.aristegui@huesped.org.ar Fundación Huésped División de Investigación en Implementación Fundación Huésped Ciudad Autónoma de Buenos Aires Argentina ines.aristegui@huesped.org.ar

**Keywords:** Personas Transgénero, Personas Travestis, Servicios de Salud para las Personas Transgénero, Siliconas, Feminización, Atención de Afirmación de Género, Argentina, Transgender Persons, Travesti Persons, Health Services for Transgender Persons, Silicones, Feminization, Gender-Affirming Care, Argentina

## Abstract

El uso de siliconas líquidas inyectables (SLI) en la población travesti y trans constituye un desafío para la salud pública de Argentina y el mundo. Aunque las SLI y otros polímeros no autorizados representan un importante riesgo para esta población, no existe un abordaje consensuado y efectivo, ni una atención de la problemática desde la política sanitaria. El objetivo del estudio fue explorar la experiencia de profesionales de la salud, de distintas especialidades y niveles de atención, en el uso, abordaje y tratamiento de las complicaciones de SLI en personas trans y travestis. Entre agosto y diciembre de 2023, se realizó un estudio cualitativo, exploratorio y descriptivo, con 14 entrevistas semiestructuradas a profesionales de la salud de cuatro provincias argentinas. Según sus relatos, las personas usuarias de SLI son una población atravesada por muchas vulneraciones, que recurre a estas prácticas debido a la inmediatez de sus resultados y a las barreras para acceder a procedimientos seguros de afirmación de género. Las y los profesionales coinciden en la no disponibilidad de información y de directrices para abordar las complicaciones del uso de SLI, especialmente en el tratamiento del dolor crónico. A su vez, se identifica el uso de SLI como un problema de salud pública, que requiere de un abordaje intersectorial e interdisciplinario.

## Abstract (en)

The use of injectable liquid silicone (ILS) in the travesti and trans population poses a public health challenge in Argentina and worldwide. Although ILS and other unauthorized polymers represent a significant risk for this population, there is no consensual and effective approach, nor has the issue been addressed through health policy. This study aimed to explore the experiences of healthcare professionals from various specialties and levels of care regarding the use, management, and treatment of ILS-related complications in trans and travesti individuals. Between August and December 2023, a qualitative, exploratory and descriptive study was carried out with 14 semi-structured interviews with health professionals from four Argentine provinces. According to their accounts, ILS users constitute a population facing multiple vulnerabilities, resorting to these practices due to the immediacy of results and the barriers to accessing safe gender-affirming procedures. Professionals agree on the lack of information and guidelines for addressing ILS-related complications, particularly in managing chronic pain. Furthermore, ILS use is identified as a public health issue requiring an intersectoral and interdisciplinary approach.

## INTRODUCCIÓN

Las siliconas líquidas inyectables (SLI) son polímeros sintéticos con una amplia gama de aplicaciones en diversas industrias, como componentes de lubricantes, adhesivos y selladores, entre otros compuestos^1^. En el ámbito de la salud, las SLI son uno de los componentes de dispositivos biomédicos, como tubos de infusión o catéteres e implantes temporales o permanentes, entre otros usos^2^. Las SLI contienen aditivos que optimizan propiedades físicas y químicas específicas, como la resistencia al calor y la flexibilidad. Estos aditivos incrementan su potencial proinflamatorio y favorecen la adherencia a los tejidos. Las SLI no son seguras para la salud y no se encuentran autorizadas para su uso como modelantes de la figura corporal por agencias regulatorias como la Food and Drug Administration (FDA)^3^. A nivel nacional, no están aprobadas por la Administración Nacional de Medicamentos, Alimentos y Tecnología Médica (ANMAT) y la Sociedad Argentina de Cirugía Plástica, Estética y Reparadora ha alertado sobre los riesgos para la salud derivados de la aplicación de SLI y desaconsejado su uso^4^. 

La prevalencia del uso de SLI en la población travesti y trans es variable de acuerdo con la región geográfica. Una encuesta realizada a 234 mujeres transgénero del área metropolitana de San Francisco reveló que una de cada dos personas transgénero había usado SLI alguna vez en la vida^5^. Los factores asociados al uso incluyen la falta de conciencia general en la comunidad acerca de los riesgos, la presión de grupo, el desarrollo de características femeninas para apoyar el trabajo sexual y la posibilidad de lograr la feminización sin la utilización de hormonas, conservando la función eréctil^5^^,^^6^. Un estudio cualitativo sobre el uso de SLI en mujeres trans y travestis identificó cuatro factores contribuyentes a su uso: una baja estima de la imagen corporal, percepciones erróneas sobre las SLI, incomodidad en espacios públicos, y un acceso limitado a seguros de salud^7^. 

En la primera encuesta en Argentina sobre población trans del año 2012, casi 8 de cada 10 encuestadas refirieron haberse inyectado siliconas alguna vez en la vida^8^. En otra investigación llevada a cabo en 2013 por Fundación Huésped y la Asociación de Travestis, Transexuales y Transgéneros de Argentina (ATTTA) se observó que el 61,2% de las personas encuestadas se habían aplicado SLI y que la gran mayoría (92,8%) lo había hecho con la ayuda de otra persona trans^9^. 

A pesar del significativo avance legislativo que representó en 2012 la promulgación de la Ley 26743 de Identidad de Género en Argentina, que garantiza el acceso a intervenciones quirúrgicas y/o tratamientos integrales hormonales para adecuar el cuerpo a la identidad de género autopercibida, las personas trans y travestis aún enfrentan barreras para acceder al sistema de salud y a prácticas e intervenciones seguras de adecuación corporal. En esta línea, Frola *et al*.^10^, mostraron una prevalencia del uso de SLI del 49,8% en una cohorte de 416 mujeres transgénero, con una media de edad de 30 años. 

La población travesti y trans en Argentina, como en otras regiones del mundo, enfrenta altos niveles de vulnerabilidad psicosocial^11^. Desde la perspectiva de Florencia Luna, la vulnerabilidad no es una condición fija o esencial, sino un fenómeno dinámico y contextual, representado a través de la metáfora de las capas. Estas capas de vulnerabilidad se superponen y pueden intensificarse debido a factores estructurales, como la discriminación, la precariedad económica y la exclusión social, pero también pueden atenuarse mediante intervenciones adecuadas. En este sentido, la población travesti y trans experimenta múltiples capas de vulnerabilidad que afectan su bienestar y su acceso a derechos fundamentales, incluyendo el derecho a la salud^12^. Los procesos de estigma y discriminación ligados a la patologización de sus identidades han tenido impactos negativos en sus condiciones de vida y en sus posibilidades de acceder a la atención médica. Por ello, en muchos casos, la vulnerabilidad en la población travesti y trans se manifiesta a través de elevados índices de depresión y otros problemas de salud mental, así como experiencias de violencia, estigma y discriminación por su identidad de género. Además, estas vulnerabilidades se ven reflejadas en los niveles educativos alcanzados, el consumo de alcohol y drogas, las dificultades de acceso a la vivienda y a la salud, entre otros factores que contribuyen a su exclusión social^13^^,^^14^. Del análisis de TransCITAR, una cohorte prospectiva de personas trans que residían en el Área Metropolitana de Buenos Aires (AMBA), se observó que, de un total de 393 mujeres transgénero con una media de edad de 32 años, el 76,3% alguna vez realizó trabajo sexual, el 50,6% no había completado los estudios secundarios, el 46,7% recibían planes de asistencia del Estado y el 42% tenían vivienda inestable^15^^,^^16^. La prevalencia de depresión en esta cohorte alcanzaba un 32,8% y el 25,4% reportó intentos de suicidio^17^. El estigma y la discriminación, junto con el bajo nivel de escolarización, la alta prevalencia del trabajo sexual y la situación de pobreza o indigencia en la que se encuentran la mayoría de las personas de la comunidad, constituyen barreras significativas para el acceso al sistema sanitario en la población travesti y trans. Un estudio realizado en 2013, en Argentina, mostró que el 41% de las mujeres trans evitan asistir al sistema de salud para no ser discriminadas^18^. En este contexto, para alcanzar el objetivo de adecuación corporal deseado, muchas personas trans y travestis recurren a SLI y otros productos similares. 

Las complicaciones asociadas al uso de SLI son múltiples y en algunos casos, como en el síndrome de embolia pulmonar, pueden ser fatales^19^^,^^20^. Entre las complicaciones más frecuentes, se encuentran la formación de nódulos subcutáneos dolorosos, la alteración local de las características de la piel y la migración del producto, que produce alteraciones estéticas y funcionales de los sitios anatómicos afectados^21^^,^^22^. También se han asociado a fenómenos de hipersensibilidad como el síndrome de ASIA y a enfermedades reumatológicas^23^. 

El abordaje de las complicaciones requiere de la evaluación de un equipo integral constituido por profesionales de distintas áreas de la salud: medicina general, medicina del dolor, neurología, reumatología, enfermedades infecciosas, salud mental, trabajo social, entre otros^24^. Sin embargo, los estudios disponibles que abordan el uso de SLI en personas trans y travestis son limitados y, en su mayoría, están constituidos por investigaciones epidemiológicas y reportes de casos de complicaciones asociadas al uso de estos productos. Las guías de práctica clínica tanto locales como internacionales^25^^,^^26^, únicamente hacen mención de su uso, pero sin profundizar en recomendaciones acerca del abordaje adecuado a las personas trans y travestis que utilizaron o quisieran utilizar SLI. Las recomendaciones disponibles están orientadas a desaconsejar el uso de SLI y a fomentar el acceso a información de calidad. No existen lineamientos claros respecto del manejo de las complicaciones derivadas de la SLI y la información disponible surge de reportes de casos, experiencias individuales y recomendaciones de expertos. 

### Enfoque integral en salud, derechos y autonomía

Nuestra investigación se enmarca dentro del trabajo conjunto que hace algunos años vienen desarrollando el Programa de Implementación de Políticas de Género y Diversidad Sexual del Ministerio de Salud, el Ministerio de Mujeres y Diversidad de la provincia de Buenos Aires y la Fundación Huesped, con el objetivo de mejorar la accesibilidad y calidad de atención de los servicios de salud para la población travesti y trans. El abordaje en salud de las instituciones que llevamos adelante este trabajo busca contribuir con la deconstrucción del “mito universalista del pensamiento científico moderno en salud que encubre quién habla y cuál es la localización epistémica de las estructuras de poder desde la cual ese sujeto universal enuncia, produce y totaliza el conocimiento”^27^. En este sentido, es necesario reconocer la salud como un campo en disputa en el que el modelo hegemónico en salud continúa limitando la salud a la (no) enfermedad, la unidad de intervención es el individuo y los colectivos se entienden como la sumatoria de individuos. La salud se reduce así a un estado estático, en el que la única búsqueda es la curación y las intervenciones se sustentan en la medicalización y medicamentalización^27^^,^^28^. Esto se encarna en el modelo de atención vigente, el modelo médico hegemónico^29^, jerarquizado por sobre otros modelos de atención con base en su anclaje en la biomedicina y en el modelo de conocimiento sostenido en la racionalidad científica. Este modelo se caracteriza también por ser biologicista, individualista, a-histórico y a-social, por lo que resulta particularmente importante su desnaturalización. Aunque el saber médico reconoce que existen otros factores como los socioculturales y económicos que afectan a las personas^30^, estas variables siempre se encuentran subjerarquizadas a las biológicas. A su vez, la definición de salud de la Organización Mundial de la Salud (OMS)^31^ como el “completo estado de bienestar físico, psíquico y social” también da cuenta del reconocimiento de otras dimensiones de la salud. Sin embargo, desde estas miradas no se comprende la salud como un proceso inherentemente social e históricamente determinado^32^, sino que “lo social” se propone como un aditivo generalmente asociado a factores de riesgo. 

Desde el análisis y reflexión de estas tensiones en el campo de la salud, nuestras instituciones se proponen trabajar por la construcción de un abordaje de la salud de manera integral, considerando al ser humano intrínsecamente como un ser biopsicosocial; es decir, como parte de una familia, una cultura, con una historia individual y social. Es así que la salud se entiende como un proceso dinámico^32^ que se desarrolla de manera particular en las distintas poblaciones, y que está determinado socialmente (por el género, la clase, la edad, el acceso a un trabajo digno, a la educación, a una vivienda, a tiempo de ocio, entre otros). Se parte de la categoría de determinación social de la salud para “descubrir la esencia del proceso de producción y distribución de la salud que la epidemiología necesita revelar”^33^. Así, la distribución desigual de la salud-enfermedad en las distintas poblaciones no se interpreta de manera individual, asociada a factores de riesgo aislados, sino que los perfiles epidemiológicos se vinculan con el sistema social. Esta categorización permite interpretar lo que se expresa de manera individual desde la determinación social de la salud y visibiliza la necesidad de construir estrategias que respondan a las particularidades de distintos grupos sociales. 

Este enfoque integral involucra la incorporación de distintas perspectivas (derechos, géneros y diversidad, entre otras). La perspectiva de derechos es un marco conceptual y de acción que promueve la autonomía de las personas y el ejercicio de sus derechos. Este enfoque interpreta las prácticas en salud más allá de la satisfacción de necesidades, y las comprende como la realización de un derecho, implicando así obligaciones y responsabilidades de distintos actores para garantizarlo^34^. En lo que respecta a la perspectiva de género permite desnaturalizar de qué manera “la construcción social y cultural de las identidades y relaciones sociales de género redunda en el modo diferencial en que hombres y mujeres pueden desarrollarse en el marco de las sociedades de pertenencia, a través de su participación en la esfera familiar, laboral, comunitaria y política”^35^. Es así que el género no es algo natural e inmutable, sino una construcción social que genera una distribución desigual de recursos y de poder e imposibilita el ejercicio pleno de los derechos humanos. La perspectiva de género permite desandar el determinismo biológico respecto a la identidad de género, e interpretar de qué manera las construcciones sociales en torno a la diferencia sexual condicionan las modalidades y formas en que las personas perciben y transitan su salud, toman decisiones respecto al cuidado, y acceden a los sistemas de atención^36^. A su vez, el modelo médico hegemónico sostiene un sistema normalizador, construyendo parámetros y sentidos sobre qué se considera normal, homologándolo con lo saludable, mientras lo que queda por fuera se constituye como patológico. En este marco de creencias, en nuestra sociedad, la heterosexualidad y la cis-sexualidad se presentan como normativas, es decir, se consideran “lo normal”. Esto se refuerza mediante el modelo médico hegemónico, en el que el prisma de inteligibilidad genera un continuo entre el sexo “biológico”, el género construido sociohistórico y culturalmente, y el deseo heterosexual^37^. La perspectiva de diversidad busca desnaturalizar esa creencia de “normatividad” para identificar y analizar las relaciones de poder y de exclusión que conlleva esta concepción de normalidad contenida en el modelo médico hegemónico, permitiendo de este modo visibilizar las experiencias singulares de las distintas comunidades, dando cuenta de los efectos negativos y las barreras de acceso constituidas desde el modelo médico hegemónico en quienes no cumplen con la “norma cis-heterosexual”. 

Como demuestran las cifras presentadas anteriormente el cis-sexismo naturalizado e invisibilizado en el sector salud^38^ es determinante de las vulnerabilidades sociales que atraviesan la comunidad travesti y trans, con impacto en su calidad de vida, en las decisiones tomadas respecto a su proceso de salud y en el acceso concreto a la atención sanitaria. Tal como fue documentado por las organizaciones travestis y trans de este país, antes de la sanción [de la Ley de Identidad de Género], las personas trans evitaban acudir a las instituciones de salud por temor a recibir maltrato y a ser víctimas de discriminación y violencia institucional^39^^,^^40^^,^^41^.

Es así que las experiencias de salud trans están atravesadas por la patologización de su identidad, proceso que se tensiona (y que se transforma) con las demandas de despatologización desde dichas organizaciones^40^^,^^41^. A su vez, la mirada interseccional permite analizar “el interjuego entre las distintas categorías de diferenciación social que atraviesan a sujetos, prácticas sociales e instituciones, y el modo en que dicho interjuego afecta a las experiencias sociales de los sujetos, su agencia política, y las relaciones de poder y oportunidades en las que se encuentran”^42^. Es así que las vivencias y experiencias de la población travesti y trans en el acceso a la salud son diversas, y deben interpretarse de manera compleja. 

Desde ambas instituciones se promueve el derecho de acceso a la salud, por lo que necesariamente se trabaja en reducir las desigualdades sociales. Para esto, se busca desarrollar investigaciones que impacten en la salud de las personas, fortalecer las organizaciones y la participación comunitaria, así como trabajar con las instituciones para desandar el modelo hegemónico en salud y construir un abordaje de la salud integral y garante de derechos. Desde estas perspectivas y en el marco de esta articulación se concluyó que era necesario contar con evidencia local actualizada sobre la problemática del uso de SLI en mujeres trans y travestis, y el abordaje de las complicaciones derivadas de su uso desde la experiencia y la mirada de efectores de salud. El objetivo del estudio consistió en explorar la experiencia de profesionales de la salud de distintas especialidades y niveles de atención, en el uso, abordaje y tratamiento de las complicaciones de SLI en personas trans y travestis, para contribuir al diseño de estrategias de información, sensibilización y capacitación de los equipos de salud de la provincia de Buenos Aires, fortalecer el trabajo de las redes territoriales e instalar la problemática en la agenda política en salud. 

## METODOLOGÍA

Se realizó un estudio exploratorio descriptivo con un enfoque cualitativo, utilizando la técnica de entrevistas semiestructuradas^46^, con el objetivo de explorar la práctica de efectores de salud que realizan atención a población travesti y trans, sobre abordajes posibles ante el uso y complicaciones de las SLI. Los participantes fueron profesionales de salud que trabajan como efectores del primer y segundo nivel de atención del subsector público de cuatro provincias argentinas (Ciudad Autónoma de Buenos Aires y provincias de Buenos Aires, Córdoba y Santa Fe) con excepción de un profesional que se desempeña actualmente en el subsector privado, aunque se formó y trabajó hasta hace algunos años en el subsector público. El criterio de inclusión fue que las y los profesionales tuvieran experiencia de atención a población travesti y trans con antecedentes de uso de SLI. El reclutamiento fue mediante la técnica de bola de nieve^47^ a partir de un listado de profesionales provisto por el Programa Provincial de Implementación de Políticas de Género y Diversidad de la provincia de Buenos Aires, complementado por el equipo de Fundación Huésped y por las personas entrevistadas. 

Entre octubre y diciembre de 2023, se realizaron 14 entrevistas en profundidad a 16 participantes (Tabla 1). Todas las entrevistas fueron individuales, a excepción de una en la que se entrevistaron tres integrantes de un mismo equipo de salud. La modalidad de las entrevistas fue virtual, exceptuando una que se desarrolló de manera presencial; con una duración aproximada de sesenta minutos. Se entrevistaron profesionales de distintas especialidades médicas incluyendo clínica médica, cirugía general, cirugía plástica, cuidados paliativos, dermatología, endocrinología, infectología, medicina general, urología, así como enfermería, trabajo social y psicología. 

**Tabla 1:** Codificación de personas entrevistadas por especialidad, subsector, nivel de atención y provincia. Argentina, agosto-diciembre, 2023.

Nombre ficticio	Especialidad	Subsector	Nivel de atención	Provincia
Ana	Infectóloga	Público	Segundo	CABA
Fabián	Cirujano	Privado	Segundo	Córdoba
José	Cirujano	Público	Segundo	Buenos Aires
Ramiro	Cirujano	Público	Segundo	Buenos Aires
Mariana	Cirujana	Público	Segundo	CABA
Luisa	Cirujana	Público	Segundo	Buenos Aires
Juana	Médica clínica	Público	Segundo	Buenos Aires
Laura	Médica generalista	Público	Primero	Buenos Aires
Maia	Médica generalista ECP	Público	Segundo	CABA
Noelia	Médica generalista ECP	Público	Primero y segundo	Buenos Aires
Julia	Dermatóloga	Público	Segundo	CABA
Celia	Licenciada en enfermería	Público	Central	Santa Fe
Lía	Médica clínica	Público	Segundo	Buenos Aires
Rocío	Trabajadora social	Público	Segundo	Buenos Aires
Lucía	Psicóloga	Público	Segundo	Buenos Aires
Roberto	Anestesiólogo	Público	Segundo	Buenos Aires
María	Endocrinóloga	Público	Segundo	CABA

Fuente: Elaboración propia. ECP= Especializada en cuidados paliativos. CABA= Ciudad Autónoma de Buenos Aires.

Se utilizó un cuestionario elaborado *ad hoc* por un equipo interdisciplinario integrado por especialistas en población travesti y trans seleccionados por la Fundación Huésped y el Programa Provincial de Implementación de Políticas de Género y Diversidad. Las preguntas que orientaron la investigación se fundamentaron en la interpretación y posicionamiento de las instituciones y poblaciones implicadas frente a problemáticas y desafíos comunes a partir de una concepción de la vulnerabilidad y exclusión de la población travesti y trans compartida por ambas instituciones. Si bien el cuestionario indagaba sobre diversos aspectos, en este artículo se focaliza en aquellos ejes de uso y en las prácticas de atención de las complicaciones de las SLI.

- Perfil de quienes consultan, motivos de consulta, especialidades médicas más consultadas y derivaciones.
- Trayectoria profesional y formación en el tema.
- Complicaciones derivadas del uso de SLI y su abordaje.
- Procedimientos para la atención de personas con complicaciones derivadas del uso de SLI.

Las entrevistas fueron grabadas, desgrabadas y analizadas temáticamente. En la medida en que se privilegió la historicidad y singularidad de los procesos y emergencias sociales, se partió de reconocer los factores y sentidos que estructuran los problemas de estudio y la manera cómo los sujetos categorizan e interpretan dichas realidades. Una vez hecho el reconocimiento de estas lógicas y significados, se acudió a referentes conceptuales y teóricos pertinentes para profundizar o problematizar la lectura inicial de los hallazgos; de este modo, el uso que se le dio a la teoría no fue deductivo (adecuar una realidad a un marco interpretativo previo) ni inductivo (“descubrir” las teorías implícitas), sino transductivo, es decir, provocar una dialéctica entre la comprensión de lo particular y la interpretación en marcos más generales, permitiendo la creación conceptual fundamentada en las prácticas sociales bajo estudio^43^^,^^44^^,^^45^.

Para la presentación de los resultados se resguardó la identidad de las personas, señalando únicamente el nombre ficticio y la disciplina y/o especialidad. El protocolo de investigación fue aprobado por el Comité de Ética del Ministerio de Salud de la provincia de Buenos Aires y el Comité de Ética de la Fundación Huésped, bajo el código de registro 11136. La investigación fue realizada por la Fundación Huésped en conjunto con el Programa de Implementación de Políticas de Género y Diversidad Sexual del Ministerio de Salud y el Ministerio de Mujeres y Diversidad de la provincia de Buenos Aires y con el financiamiento del Consejo Federal de Inversiones de Argentina (EX-2023-00068948-CFI-GES#DC). Cada una de las personas participantes firmó un consentimiento informado previo a la realización de las entrevistas.

## RESULTADOS

### Perfil de quienes consultan, motivos de consulta, especialidades médicas más consultadas y derivaciones

Dentro de la población que consulta por complicaciones a causa del uso de SLI, las personas entrevistadas distinguieron dos grupos de acuerdo con el género y la edad. Respecto al género, las mujeres trans y travestis fueron consideradas la principal población afectada, la que vive en las peores condiciones, con un deterioro importante de su salud física y mental que, según algunas entrevistas, habría mejorado si se la compara con una década atrás. Según las y los profesionales, las mujeres trans y travestis recurren al trabajo sexual como principal medio de vida y, en el caso de las adolescentes más jóvenes, a la creación de “contenido sexual” para sitios de Internet. Poseen un bajo nivel educativo si se las compara con la población de varones trans o personas no binarias, y tienen la particularidad de integrar redes de cuidado que son el fruto de su organización y movilización política y del acompañamiento de algunas políticas públicas del último decenio.

“*La mayoría de las* [compañeras trans y travestis] *que acompañamos son compañeras que no están alfabetizadas o están alfabetizadas de manera precaria a través de redes como el Whatsapp; son migrantes, la mayoría de las que acompañamos son de Perú, y la mayoría se dedican al trabajo sexual como su actividad principal que son las usuarias de siliconas líquidas*”*.* (Juana, médica clínica)

Algunas de las personas profesionales entrevistadas, en su mayoría del área de cirugía, reportaron que esta problemática también afectaba a mujeres cis. Con relación a la edad, el promedio de las mujeres trans y travestis que usaron SLI varía según el perfil de la persona entrevistada y el servicio. En algunos casos se hizo referencia a edades entre 20 y 30 años, y en otros entre los 40 y 50 años. Este último grupo etario fue calificado en las entrevistas como una edad avanzada para la expectativa de vida promedio de una mujer trans o travesti. Algunas personas entrevistadas señalaron que actualmente observaban una disminución en el uso de SLI en las mujeres trans y travestis más jóvenes, alertadas por la experiencia de las generaciones previas. Otras personas refirieron que las más jóvenes y recién llegadas al AMBA desde otras provincias de Argentina (sobre todo del noroeste argentino) y ciudades del interior del país como Rosario o de países limítrofes, continúan recurriendo al uso de SLI. Los motivos mencionados fueron la falta de información y de acompañamiento, y porque no tienen otra alternativa para poder incorporarse rápidamente al trabajo sexual como medio de subsistencia. En algunas entrevistas se destacó que la población de varones trans y personas no binarias es más joven, con mayor nivel educativo, incluso universitario, y empleos más estables y mejor pagos con respecto a la población de mujeres trans y travestis. Solo en una entrevista se refirieron a un caso de atención de un varón trans que se había colocado siliconas - “aceite de camión” - en glúteos y pectorales. 

La edad de la población también se relacionó con el contexto histórico en el que la persona inició su proceso de adecuación corporal o de afirmación de género. Varias personas consultadas aluden a que la Ley 26743 de Identidad de Género, la Ley 27636 de Acceso al Empleo Formal para personas Travestis, Transexuales y Transgéneros -Cupo laboral Travesti Trans- y los programas y dispositivos de políticas públicas derivados de estas normas, funcionan como determinantes contextuales de un mayor acceso a la información, orientación, servicios y tratamientos de afirmación de género para las personas más jóvenes.

“*Son les pibis que son hijes de la ley, que ya vienen solicitando cosas específicas, ya vienen con un recorrido hecho*”. (Laura, médica generalista)

Las y los profesionales identifican el acceso a la hormonización como la opción segura para la afirmación de género y/o como paso previo a la cirugía de afirmación de género en aquellos casos en que esta es deseada. En lo que respecta al vínculo de la población travesti y trans con los servicios de salud, en las entrevistas se refirieron a problemas de acceso, un vínculo errático con los servicios, y antecedentes de maltrato y discriminación reiterados que explican que estas personas desistan muy rápidamente de la consulta si no obtienen soluciones efectivas. Su recorrido por los servicios, sumado al dolor crónico e inhabilitante que no logran manejar, hace que se sientan frustradas, desilusionadas, desgastadas y cansadas de seguir adelante.

“*Es muy complejo porque la compa está en una situación de vulnerabilidad, a veces con escasez de alimentos, es trabajadora sexual, es un lío. No la vemos a veces por largas temporadas entonces cuando vuelve suele volver con alguna complicación*. […] *Han sido ninguneadas y maltratadas durante toda su vida así que no vienen muy entusiastas a la consulta, sigue siendo un escenario complejo de acompañamiento*”*.* (Juana, médica clínica)

En la Tabla 2 se presentan las especialidades, motivos de consulta y derivaciones o interconsultas más frecuentes referidos por las personas entrevistadas. 

**Tabla 2:** Especialidades más consultadas, motivos de consulta y derivaciones, según las y los profesionales entrevistados de cuatro provincias argentinas. Agosto-diciembre, 2023.

Especialidades más consultadas	Motivos de consulta	Derivaciones
Cirugía	Retiro de las SLI que han migrado y generado deformidades con dolor, inflamación e infecciones.	Reumatología para que maneje o mitigue la reacción a la presencia del material extraño.
Clínica médica, medicina general, medicina familiar	Dolor, inflamación o infección aguda que motiva consulta en guardia, o dolor e inflamación crónicos que aparecen en el contexto de una consulta de salud integral asociada al acompañamiento sobre todo de infecciones de transmisión sexual (ITS); o usuarias que viven con VIH, hepatitis B, o tienen diagnóstico de sífilis.	Cirugía, dermatología, traumatología de miembros inferiores, salud mental. Medicina del dolor, reumatología, trabajo social.
Infectología	Infecciones de transmisión de sexual	Reumatología, traumatología (sobre todo de miembros inferiores).
Endocrinología	Dolor e inflamación crónicos que aparecen en el contexto de una consulta en el marco de hormonización.	Salud mental (debido a situaciones de consumo problemático, de depresión o para el manejo del dolor crónico) y clínica médica
Dermatología	Alteraciones en el aspecto y color de la piel. Proceso inflamatorio crónico a nivel de la piel con aparición de granulomas y de procesos cicatrizales crónicos que producen una alteración trófica en la piel, que la hace más propensa a infecciones.	Salud mental (debido a situaciones de consumo problemático, de depresión o para el manejo del dolor crónico), cirugía y clínica médica.

Fuente: Elaboración propia.

### Trayectoria profesional y formación en el tema

Las personas entrevistadas coincidieron en que su formación en atención de población travesti, trans y no binaria usuaria de SLI era resultado de su iniciativa autodidacta, su experiencia de trabajo como efectores de salud o programas dentro de fundaciones o gobierno, o del intercambio con pares con trayectoria en la cuestión, y el diálogo de saberes con personas usuarias de los servicios. Otra fuente de conocimiento que refirieron fueron congresos médicos donde pudieron acceder a protocolos aplicados en otros países.

“*De silicona líquida si hay alguna capacitación avísenme, porque nunca me enteré que existiera. De hecho, lo que empezó a pasar cuando empecé a estar más activamente dentro del consultorio, es que las situaciones que iban apareciendo, y para las cuales yo no tenía respuesta ni formación, preguntar al Programa* [...] *Es como una búsqueda de hormiga y hasta ahora yo no siento que esté saldada esa búsqueda*”. (Laura, médica generalista).

Se destacó que la formación dentro de Argentina consistió en cursos cortos y específicos, en su mayoría sobre hormonización o diplomaturas sobre enfoque de género y diversidad en salud y no eran específicos del uso y complicaciones de las SLI. Varias personas entrevistadas señalaron el Programa de Políticas de Género y Diversidad del Ministerio de Salud de la provincia de Buenos Aires. Las y los profesionales de cirugía merecen una mención particular ya que la especialización en el trabajo con la población travesti y trans, con antecedentes de uso de SLI, fue referida en varios casos como el resultado del “azar” o de un “accidente” a partir del contacto con profesionales y posgrados en el exterior referentes en materia de cirugía plástica en esa población.

“*La verdad que fue de casualidad el hallazgo digamos de atención a la población travesti, trans y no binaria,* [...] *cuando yo hice el fellow de feminización facial yo no lo tenía previsto, no era algo que yo había buscado sino que bueno… circunstancias de la vida*...”. (Fabian, cirujano)

### Complicaciones derivadas del uso de SLI y su abordaje

En cuanto a las complicaciones, las entrevistas confirman la información disponible en los estudios citados en este trabajo. Si bien existen consecuencias graves e incluso letales a corto plazo, las más frecuentes son aquellas a mediano y largo plazo. Estas incluyen la formación de siliconomas que migran, causan deformidades, episodios agudos de dolor, inflamación o sobreinfección. Los siliconomas son frecuentes y se agravan con el tiempo, limitando la movilidad, causando dolor y deteriorando la calidad de vida.

“*Así que el recuerdo que tengo es de mucho sufrimiento de este paciente. Y mucha culpa*”. (Maia, médica generalista especialista en cuidados paliativos)

Un equipo de cuidados paliativos especificó:

*“Lo que puede verse más frecuente es que migra por todo el cuerpo y se enquista y genera mucho dolor, es el dolor crónico lo que molesta en el correr del tiempo, porque se diluye, porque se palpa, porque genera una sensación de disconfort y de dolor.* [...] [Y reforzó comentando] *La primera consulta es el dolor. Un malestar absolutamente inhabilitante por más que el umbral del dolor cambie para cada una, en muchos casos es inhabilitante. Después todo lo otro que conlleva la cuestión fisiológica, la cuestión pulmonar o renal, otras cuestiones que aparecen después, pero las consultas fueron específicamente por el dolor*”. (Lia, médica clínica)

Algunas de las personas entrevistadas, en su mayoría del área de cirugía, advirtieron también sobre la alta prevalencia de complicaciones derivadas del uso de SLI en personas cisgénero, sobre todo mujeres. En la Tabla 3 se presentan sintéticamente las complicaciones que motivan la consulta. 

**Tabla 3:** Complicaciones que motivaron la consulta médica, según las y los profesionales entrevistados de cuatro provincias argentinas. Agosto-diciembre, 2023.

Complicaciones locales	Complicaciones sistémicas
Discromías	Migración y complicaciones a distancia
Hematomas	Neumonitis
Eritema	Síndrome de distrés respiratorio
Edema	Hepatitis granulomatosa
Cambios en la textura de la piel	Síndrome de embolia por SL-TEP
Granulomas	Infecciones generalizadas
Fístulas	Granulomas por cuerpo extraño
Úlceras	Enfermedades autoinmunes
Infecciones locales	Enfermedades del tejido conectivo
	Síndrome de ASIA

Fuente: Elaboración propia.

Dado que la extracción parcial o total de las SLI no siempre es posible, un abordaje paliativo es frecuente, para tratar la infección recurrente, reducir la inflamación y/o aliviar el dolor crónico en función de la situación y la evolución de cada persona. Resulta muy importante el manejo del padecimiento crónico, disminuir la reactividad al cuerpo extraño, la educación en el reconocimiento de alertas y la incorporación de hábitos para disminuir las complicaciones o evitar que se agraven. Las cirugías ablativas y reconstructivas no siempre son factibles de realizar por las condiciones en las que se encuentra la persona. Pueden no ser convenientes por lo mutilante de la intervención y la extracción total del material es poco probable. Desde algunas especialidades aconsejan que, si la persona aún no manifiesta complicaciones, síntomas o molestias, “*es mejor no innovar*”. Para otras, la cirugía es una opción adecuada y conveniente en ciertos casos como cuando los siliconomas generan muchas complicaciones y/o son invalidantes, o se ubican en ciertas zonas del cuerpo (en especial mamas) y no están adheridos a otras partes del cuerpo.

“*En algunos casos si está bien localizado y tenés nódulos, entonces podes acceder a ellos y sacas cada uno de esos nódulos, pero cuando está desparramado, le ofreces la posibilidad de sacarle la mayor cantidad posible, pero no es la totalidad*”. (Luisa, cirujana)

Las personas entrevistadas sugieren tomar recaudos, valorar junto a quien consulta los riesgos y las probabilidades de éxito de las intervenciones y decidir en conjunto la conducta a seguir. Las y los profesionales recomiendan indicar en las consultas las siguientes pautas de cuidado: 1) evitar el calor, en especial a nivel local; 2) evitar ejercicios de alto impacto; 3) prevenir el trauma directo sobre la zona afectada; 4) evitar tratamientos estéticos en zonas que han sido inyectadas con sustancias de relleno; 5) evitar tratamientos que promueven la disolución, punción, aspiración, y rotura de vesículas; 6) evitar largos periodos de apoyo sobre las zonas afectadas; 7) evitar masajes o drenaje linfático; 8) evitar ropa ajustada que provoque fricción sobre la piel; 9) evitar cualquier tipo de punción-inyección en las áreas afectadas (en caso de glúteos, usar en su lugar el muslo o la zona deltoidea); y 10) mantener un peso equilibrado, ya que las fluctuaciones pueden favorecer la migración de las siliconas líquidas. 

### Procedimientos para la atención de personas con complicaciones derivadas del uso de SLI

Las personas entrevistadas coinciden en que no existen protocolos de atención para quienes tienen antecedentes de uso de SLI, aunque sí algunos procedimientos para el abordaje de complicaciones específicas que los equipos fueron aprendiendo a través del tiempo y durante su formación en el servicio o tras la interacción con personas usuarias. Las y los profesionales que trabajan en consultorios especializados en población travesti y trans y/o que integran equipos interdisciplinarios sí pudieron dar una visión más integral o completa y proponer un abordaje coherente con esa mirada. Al momento de realizar la anamnesis y confeccionar la historia clínica, las personas entrevistadas resaltan la importancia de indagar sobre los antecedentes de la persona, su identidad de género autopercibida y el nombre con el que quiere ser llamada, como también preguntar sobre la expectativa y/o necesidad que motiva la consulta. Asimismo, destacan explorar cuál es el conocimiento previo respecto al uso de SLI: cuándo se realizó, con qué material, y dónde se realizó el procedimiento. Es importante conocer si la inyección fue realizada por profesionales de la salud o si fueron personas no capacitadas y cuáles fueron las primeras consecuencias del uso de SLI que registraron. En esta instancia es importante fortalecer el registro del propio cuerpo, para identificar posibles complicaciones de manera temprana y consultar al equipo de salud e indagar sobre otros procedimientos de cambios corporales como la hormonización o implantes mamarios, aconsejando a las personas a optar por las prácticas más seguras.

Por tratarse de una práctica desaconsejada desde los equipos de salud es muy probable que las personas con antecedentes de uso de SLI no lo refieran de forma espontánea, sobre todo en una primera consulta, por lo que las personas entrevistadas insistieron en la importancia de generar un clima de confianza, en el que la persona no se sienta criminalizada y preguntar específicamente sobre el uso de SLI, explicando el porqué de dichas preguntas: “*no quieren contar, porque muchas veces saben que es una práctica que dentro de lo médico no es una práctica que esté validada*” (Ana, infectóloga). De las entrevistas surge que la mayoría de las personas que usaron SLI no sabe qué fue lo que se les inyectó y en qué cantidad. Una de las profesionales entrevistadas puntualizó:

“…*por ejemplo el metacrilato, el subiton, el PMMA, el polimetilmetacrilato, o sea, son materiales que son más sólidos. Entonces, cuando el paciente viene con un nódulo puntual, es más fácil de tratar, que cuando viene con una inyección de material líquido*”. (Mariana, cirujana)

En algunas entrevistas se señaló que primero debe establecerse si la persona está en fase aguda, crónica o sin complicaciones evidentes:

“…*si el paciente está en fase aguda, por ejemplo, por un proceso inflamatorio o infeccioso* [que constituya una emergencia o situación de riesgo para su vida; o se trata de] *un paciente en fase crónica, que no viene con un problema agudo, que se inyectó hace un montón de años y empiezan las secuelas de la inyección* […] *nódulos, dolor, deformidades, problemas en el drenaje linfático, linfangitis, alteraciones en la circulación, alteraciones en la calidad de la piel, en el color de la piel* [o si se trata de personas sin complicaciones evidentes que están] *asustadas por todo lo que están escuchando y viendo por televisión y quieren saber qué tienen puesto, quieren saber qué les va a pasar, si tiene consecuencias*”. (Mariana, cirujana)

Las personas consultadas señalaron que es importante indagar si hubo golpes o eventos recientes que expliquen la fase aguda porque “*el siliconoma está tranquilo y por algún motivo, un golpe, una vacuna que se dieron, un cuadro viral, se inflama y se reactiva*” (Julia, dermatóloga).

Además del examen clínico, las tecnologías de diagnóstico por imágenes más utilizadas para el abordaje de las complicaciones por el uso de SLI son la ecografía, la mamografía, la tomografía computada y principalmente la resonancia magnética. Sobre esta última, algunas personas refirieron dificultades para el acceso a los turnos. Al examen clínico y el diagnóstico por imágenes, se suma el laboratorio para evaluar la función renal, hepática y hormonal. En varias entrevistas se planteó la conveniencia de la interconsulta con reumatología para detectar en forma temprana una enfermedad autoinmune asociada o para evaluar la necesidad de medicación que contrarreste la respuesta exagerada del organismo a la presencia de las SLI como material extraño.

“*Y muchas veces la interconsulta con reumato y* [...] *evaluar si hay un proceso inflamatorio crónico en el organismo, porque si es así, a veces es necesario bajarlo, hay que enfriar esos pacientes*”. (Mariana, cirujana)

Como ya se planteó, las poblaciones usuarias de SLI se encuentran en situación de vulnerabilidad y ejercen el trabajo sexual por lo que están más expuestas a adquirir infecciones de transmisión sexual. Por ello, en algunos casos se deriva a infectología para diagnosticar y tratar, si en el examen clínico se observó alguna lesión, o se deriva a ginecología o urología si se observan verrugas. Debido a que la población usuaria muy a menudo realiza tratamientos de hormonización sin supervisión médica o presenta consumo problemático de sustancias o cuadros de depresión, se identificó la oportunidad de proponerle a la persona una interconsulta o articulación con endocrinología y salud mental respectivamente, en pos de mejorar el acceso a la atención en salud. La articulación con servicios de salud mental también se debía al acompañamiento del dolor crónico, que muchas veces conlleva el uso de las SLI, y a su impacto en la vida cotidiana de las personas.

De acuerdo con las entrevistas, la mayoría de los tratamientos tienen un propósito paliativo y no curativo:

*“Es que están muy limitadas las intervenciones. Generalmente, los tratamientos que van a realizarse van a ser para lo que te contaba, para paliar algún síntoma*”. (Maia, médica generalista especialista en cuidados paliativos)

Las consultas se centran en tratar la infección recurrente, bajar la inflamación y/o calmar el dolor crónico según lo requiera. Por esto resulta más importante el manejo del padecimiento crónico, disminuir la reactividad al cuerpo extraño, el reconocimiento de alertas, y la incorporación de hábitos que prevengan la aparición o agraven las complicaciones ya existentes. Asimismo, varios profesionales destacaron la importancia de tranquilizar, aconsejar y acompañar a las personas, principalmente, a las que padecen un cuadro crónico sin que se vislumbre la posibilidad de una intervención quirúrgica conveniente.

“*...tratamos de que no haya más complicaciones a futuro enfriando el proceso y tratando de explicarles que a veces la extracción es quirúrgicamente imposible* [...] *todas esas son cosas que hablamos con los pacientes y consensuamos a ver cuál es el mejor tratamiento posible*”. (Mariana, cirujana)

## DISCUSIÓN

Nuestro estudio es el primero en explorar y sistematizar la experiencia de profesionales de la salud de distintas especialidades en el abordaje y tratamiento de las complicaciones por uso de SLI en Argentina. Nos permitió no solo entender prácticas actuales de atención, sino también comprender en profundidad algunos aspectos vinculados a la población travesti y trans y al modo de uso de las SLI, y el tipo de vinculación de la comunidad con el sistema de salud en esta problemática en particular.

De las entrevistas se desprende que la población usuaria de SLI presenta un alto grado de vulnerabilidad social, destacando la alta tasa de personas migrantes que ejercen el trabajo sexual de subsistencia, con grupos etarios que oscilan entre los 20 a 50 años. Para facilitar el acceso a la salud de esta población excluida es necesario contar con dispositivos específicos y adaptados que brinden una atención alineada con las necesidades de esta comunidad. Particularmente, se destaca el contar con horarios de atención ampliados, especialmente para aquellas personas que ejercen el trabajo sexual, disminuir la excesiva burocracia en la atención, y contar con personal entrenado en materia de género, que asegure una atención que respete los derechos de las personas trans^48^. La Organización Panamericana de la Salud propone en sus directrices^49^, la creación de servicios de atención integrales que provean variadas herramientas de prevención y tratamiento de ITS (preservativos, lubricantes, servicios de detección y medicación, entre otras), asociadas al ofrecimiento de tratamientos de hormonización y asesoramiento sobre otros aspectos importantes para la comunidad trans, como los problemas en salud mental o el consumo problemático de sustancias. En la misma línea, los resultados de las entrevistas muestran que un elemento esencial para garantizar una atención integral a la población travesti y trans es el acceso a procedimientos médicos seguros que les permitan adecuar su apariencia física al género autopercibido, como la terapia de hormonización y procedimientos quirúrgicos de afirmación de género. Sin embargo, la situación actual que se expone en este estudio, y que se encuentra en línea con investigaciones previas^8^^,^^9^^,^^10^^,^^18^, indica que el uso de SLI para la modificación corporal es una práctica frecuente y peligrosa que afecta de forma particular a la comunidad de mujeres trans y travestis, que se realiza en condiciones precarias y por personal no capacitado, utilizando productos no aptos para uso en el cuerpo. Desde la óptica de las y los profesionales, lo que motiva a las personas a optar por las SLI es su deseo y necesidad de modificación corporal inmediata, a un costo accesible, en un contexto en el que otras alternativas no son factibles, viables o rápidas. Aunque con el correr de los años se dispone de más información sobre SLI y sus riesgos para la salud, para las personas más excluidas -de redes de cuidado y medios para la superveniencia- continúa siendo una necesidad priorizar el resultado por sobre posibles complicaciones futuras, que además no siempre son evidentes o conocidas entre pares.

Las complicaciones derivadas del uso de SLI representan un terreno poco conocido y con escaso sustento bibliográfico y los profesionales que atienden a las personas afectadas no cuentan con directrices claras que los guíen en su práctica. Las y los profesionales entrevistados concordaron en que nunca recibieron formación específica para el abordaje de complicaciones por uso de SLI, y manifestaron no conocer protocolos ni guías de atención al respecto. Solo hicieron referencia a algunos procedimientos que fueron encontrando con el tiempo, y aprendiendo durante su formación en el servicio y en la interacción con las personas usuarias. Esto se sustenta en la escasa información disponible sobre este tema. Diversas guías nacionales e internacionales que abordan temáticas de salud importantes para la comunidad trans, o bien omiten el tema o realizan una somera descripción^16^^,^^25^. Recientemente, la Organización Mundial de la Salud (OMS) realizó un llamado de expertos a nivel global para conformar un panel, a fin de confeccionar las nuevas guías de salud trans, que pondrán foco en diversos aspectos, que incluyen la prestación de cuidados para la afirmación de género, educación y formación del personal sanitario, abordaje de situaciones de violencia, políticas sanitarias y reconocimiento legal de la identidad de género^26^. 

Para poder abordar las complicaciones del uso de las SLI, los equipos deben contar con formaciones específicas en esta temática, así como deben contar con miradas de múltiples áreas, como cirugía, clínica médica, cuidados paliativos, dermatología, medicina general, medicina de emergencias, reumatología, infectología, salud mental y trabajo social^24^. Al ser una problemática tan compleja, resulta necesario el armado de redes de derivaciones que permitan un seguimiento de la persona y una articulación entre los distintos servicios y niveles de atención involucrados. Durante las entrevistas quedó expuesta esa necesidad de disponer de información de calidad para poder dar respuestas a esta demanda de salud insatisfecha. Es así que resulta necesario profundizar el trabajo intersectorial -entre el nivel central, el sector salud, la sociedad civil y las organizaciones sociales- para construir un nuevo conocimiento.

Las personas entrevistadas enumeraron algunas de las complicaciones agudas y crónicas derivadas del uso de SLI, que van en línea con los reportes hallados en la bibliografía^19^^,^^20^^,^^21^^,^^22^^,^^23^. En lo que respecta al manejo de los siliconomas o los nódulos producidos por la SLI, no hay un consenso en la bibliografía^24^, y las y los profesionales de cirugía reflejan estas ópticas dispares, que van desde ser conservadores a recomendar la cirugía en ciertas ocasiones para eliminar parte del material extraño. Estas complicaciones comprometen la calidad de vida de las personas afectadas, quienes están obligadas a consultar en forma permanente a los servicios de salud para tratar los síntomas recurrentes derivados del uso de SLI. 

Por otro lado, coinciden en la importancia de manejar el padecimiento crónico, disminuir la reactividad al cuerpo extraño, entrenar a la persona para reconocer situaciones de alarma y para que incorpore hábitos que disminuyan la posibilidad de activación de las SLI. El abordaje de padecimientos crónicos y complejos constituye un desafío para todas las personas profesionales de la salud y, en particular, para quienes atienden a la población travesti y trans. El modelo hegemónico en salud actual no incorpora fácilmente enfoques interdisciplinarios ni provee el tiempo o la formación necesaria para escuchar activamente a las personas, establecer una relación profesional-consultante basada en la confianza, entender las necesidades de las personas usuarias y considerar sus miradas en el armado del plan de acción. Las y los profesionales consultados indican que la escucha activa, el cultivo de la confianza y el enfoque centrado en la persona son cruciales para comprender la experiencia del dolor crónico de manera integral. Las guías de prácticas clínicas recomiendan crear un ambiente de respeto, habilitando un espacio de diálogo e intercambio^25^, ya que esto favorece el seguimiento, la adherencia a los tratamientos y, en consecuencia, la respuesta a los tratamientos. En este sentido, el armado de consultorios específicos para esta problemática, que se lleven adelante de manera interdisciplinaria y permitan ir construyendo otras formas y modos de atención en salud para el abordaje de situaciones complejas puede constituir una estrategia eficaz. Estos nuevos modos de atención deben incorporar también la participación de la comunidad, que posee un saber específico sobre esta temática y también puede favorecer el acceso y disponibilidad del efector de salud para con las personas trans y travestis^49^. Por otro lado, la relevancia del abordaje paliativo de las complicaciones de las SLI desafía a los equipos a formarse en enfoques no farmacológicos e interdisciplinarios para el manejo del dolor crónico. En este sentido, el trabajo entre fisioterapeutas, psicólogos y trabajadores sociales, entre otras especialidades, puede derivar en mejores resultados terapéuticos para proporcionar una atención integral a las personas con dolor crónico. Del mismo modo, la prescripción prudente de analgésicos tradicionales, opiáceos u otras alternativas farmacológicas disponibles y su uso racional por parte de la población constituyen desafíos de los equipos que trabajan con la población usuaria de SLI. Asimismo, resultan importantes las intervenciones no farmacológicas que abordan aspectos físicos, emocionales y sociales del dolor crónico, tanto como las iniciativas para garantizar el acceso equitativo a tratamientos efectivos y cobertura integral de servicios de salud mental, programas de rehabilitación física y medidas para abordar los determinantes sociales que pueden exacerbar el dolor crónico. 

### Alcances y limitaciones del estudio

Este estudio aporta evidencia basada en la práctica de equipos asistenciales con experiencia de atención a población travesti y trans sobre un problema de salud pública prevalente, que se encuentra invisibilizado y sobre el que no se conocen estudios que permitan orientar la atención. Es fundamental que el tema sea discutido y se instrumenten estrategias para abordar el uso de SLI y sus complicaciones. En este sentido, este estudio intenta poner en agenda la temática, brindando un amplio abanico de percepciones y recuperando las experiencias de profesionales que intentan responder algunos de los aspectos del tema, teniendo en cuenta la escasa información publicada disponible. 

Dentro de las limitaciones del estudio podemos mencionar que se utilizó un muestreo no probabilístico con un posible sesgo de muestreo, ya que las redes alcanzadas tienden a compartir características o experiencias similares. A su vez, la cantidad de personas entrevistadas en este estudio puede considerarse una limitación en la suficiente información y experiencias recolectadas; sin embargo, representa un paso importante para la construcción de conocimiento en esta área invisibilizada. Para contrarrestar este sesgo, desde un principio se buscó tener una muestra con la mayor diversidad posible en cuanto a niveles de atención y a especialidades, focalizando en especialidades médicas que aborden la problemática de las SLI y logrando una diversidad de trayectorias y puntos de vista dentro de esta disciplina (la medicina). En la muestra hubo una mayoría de personas entrevistadas de cirugía y de medicina general, frente a una menor representación de otras especialidades. 

Al realizarse entrevistas semiestructuradas, la generalización de resultados podría ser una limitación del estudio. Aunque excede el objetivo, la triangulación de datos con cuestionarios cuantitativos o con entrevistas a personas que viven con SLI en sus cuerpos, sin duda enriquecería los resultados y será una propuesta a retomar en futuras intervenciones. A su vez, recuperar la voces y experiencias de los equipos de salud es parte del posicionamiento de las instituciones que llevaron adelante el estudio.

### CONCLUSIONES

La indagación y sistematización de prácticas actuales en torno al abordaje del uso y tratamiento de las complicaciones de SLI en la población travesti y trans resultó clave para identificar algunos puntos sobresalientes para avanzar en acciones concretas que mejoren las intervenciones en salud. Por un lado, el uso de SLI constituye un problema de salud pública que es necesario poner en la agenda de los tomadores de decisión para ser tenido en cuenta. Si bien existe una mayor disponibilidad de información en lo que respecta a las complicaciones del uso y un mejor acceso a otras prácticas seguras de afirmación de género, se identifica que la aplicación de SLI continúa siendo una práctica frecuente, en particular, en personas travestis y trans jóvenes y migrantes (migración tanto interna como externa). Esta situación se ve atravesada por estándares de belleza corporal y normas sociales que, en el caso de las personas que se dedican al trabajo sexual, es prácticamente un requisito para su ejercicio. A su vez, se agrava por la persistencia de barreras de acceso de la población a servicios adecuados y a programas de hormonización e implantes y prácticas más seguras de afirmación de género. Es así como las determinaciones sociales de la salud forman parte estructural de esta problemática y deben ser tenidas en cuenta al momento del armado de protocolos de atención o guías de prácticas clínicas y en la implementación de políticas de salud. Queda en evidencia en el relato de las personas entrevistadas el impacto positivo de normativas como la Ley de Identidad de Género y el Cupo Laboral Travesti Trans y los programas y dispositivos de política pública derivados de estas normas, que garantizan un mayor acceso a información, orientación, servicios y tratamientos en la vida de las personas travestis y trans. En este sentido, también se puede resaltar que de las entrevistas surgen acciones que quedan por hacer para todas las generaciones de personas travestis y trans. 

Por otro lado, identificamos que hay una demanda concreta por parte de profesionales de la salud de realizar formaciones específicas en abordaje de las complicaciones de las SLI. Las personas travestis y trans con SLI tienen diversas puertas de entrada al sistema de salud: guardia, consultorios de diversidad, endocrinología, infectología, cirugía, trabajo social, salud mental, entre otras. Actualmente, los equipos de salud abordan estas complicaciones, y se van capacitando en la práctica o de manera autodidacta, pero no cuentan con espacios de formación o discusión formales y no existe un lineamiento claro o protocolo que enmarque sus intervenciones. 

Es necesario generar más investigaciones sobre el impacto del uso de SLI en la salud de las mujeres trans y travestis y desarrollar estrategias intersectoriales para prevenir esta práctica, a la vez de hacer disponibles para la comunidad alternativas seguras y efectivas para la afirmación de género. Asimismo, es fundamental promover el acercamiento y la vinculación del sistema de salud con la comunidad travesti y trans, desandar el modelo médico hegemónico y sus implicancias para garantizar una atención respetuosa, inclusiva y transcompetente
